# Severe neonatal onset neuroregression with paroxysmal dystonia and apnoea: Expanding the phenotypic and genotypic spectrum of *CARS2*‐related mitochondrial disease

**DOI:** 10.1002/jmd2.12360

**Published:** 2023-01-22

**Authors:** Jessie Poquérusse, Melinda Nolan, David R. Thorburn, Johan L. K. Van Hove, Marisa W. Friederich, Donald R. Love, Juliet Taylor, Christopher A. Powell, Michal Minczuk, Russell G. Snell, Klaus Lehnert, Emma Glamuzina, Jessie C. Jacobsen

**Affiliations:** ^1^ School of Biological Sciences The University of Auckland Auckland New Zealand; ^2^ Centre for Brain Research The University of Auckland Auckland New Zealand; ^3^ Department of Neurology Starship Children's Health Auckland New Zealand; ^4^ Murdoch Children's Research Institute Melbourne Victoria Australia; ^5^ Department of Paediatrics The University of Melbourne Melbourne Victoria Australia; ^6^ Department of Pediatrics, School of Medicine University of Colorado Anschutz Medical Campus Aurora Colorado USA; ^7^ Department of Pathology and Laboratory Medicine Children's Hospital Colorado Aurora Colorado USA; ^8^ Diagnostic Genetics LabPLUS, Auckland City Hospital Auckland New Zealand; ^9^ Genetic Health Service New Zealand Auckland City Hospital Auckland New Zealand; ^10^ MRC Mitochondrial Biology Unit University of Cambridge Cambridge UK; ^11^ Adult and Paediatric National Metabolic Service Auckland City Hospital Auckland New Zealand; ^12^ Present address: Division Chief, Pathology Genetics Sidra Medicine Doha Qatar

**Keywords:** CARS2, mitochondrial disorders, neurodevelopmental disorder, tRNA synthetases, whole‐exome sequencing

## Abstract

Disorders of mitochondrial function are a collectively common group of genetic diseases in which deficits in core mitochondrial translation machinery, including aminoacyl tRNA synthetases, are key players. Biallelic variants in the *CARS2* gene (NM_024537.4), which encodes the mitochondrial aminoacyl‐tRNA synthetase for cysteine (CARS2, mt‐aaRS^cys^; MIM*612800), result in childhood onset epileptic encephalopathy and complex movement disorder with combined oxidative phosphorylation deficiency (MIM#616672). Prior to this report, eight unique pathogenic variants in the *CARS2* gene had been reported in seven individuals. Here, we describe a male who presented in the third week of life with apnoea. He rapidly deteriorated with paroxysmal dystonic crises and apnoea resulting in death at 16 weeks. He had no evidence of seizure activity or multisystem disease and had normal brain imaging. Skeletal muscle biopsy revealed a combined disorder of oxidative phosphorylation. Whole‐exome sequencing identified biallelic variants in the *CARS2* gene: one novel (c.1478T>C, p.Phe493Ser), and one previously reported (c.655G>A, p.Ala219Thr; rs727505361). Northern blot analysis of RNA isolated from the patient's fibroblasts confirmed a clear defect in aminoacylation of the mitochondrial tRNA for cysteine (mt‐tRNA^Cys^). To our knowledge, this is the earliest reported case of CARS2 deficiency with severe, early onset dystonia and apnoea, without epilepsy.

**Synopsis**

Biallelic pathogenic variants in the *CARS2* gene are associated with lethal neonatal dystonia and apnoea.

## 1 INTRODUCTION

Disorders of mitochondrial function are a collectively common group of genetic disorders with a prevalence rate reaching 1:5000 to 1:10 000 live births.^1^ The disorders are caused by pathogenic variants in a wide range of both mitochondrial‐ and nuclear‐encoded genes and manifest in the form of multisystemic phenotypes of varying onset and severity. The aminoacyl‐tRNA synthetases (aaRSs) are a family of proteins essential to the initiation of translation and production of cytosolic and mitochondrial proteins, via the catalysis of attaching an amino acid to its cognate tRNA. There are 38 distinct cytoplasmic and mitochondrial aaRS genes, all encoded by nuclear DNA. The primary role of aaRS proteins is the aminoacylation of tRNAs and translational proof reading, but secondary roles also exist.^2^ Biallelic pathogenic variants in the genes encoding mitochondrial aaRS (mt‐aaRS) yield mitochondrial protein translation‐specific disorders with clinical phenotypes spanning infantile cardiomyopathy (*AARS2*),^3^ various encephalopathies (*CARS2*, *DARS2*, *EARS2*, *MARS2*, *FARS2*, *RARS2*),^4^, ^5^ renal failure (*SARS2*),^6^ myopathy with sideroblastic anemia (*YARS2*),^7^ and ovarian failure and hearing loss (*HARS2*, *LARS2*).^8^

The *CARS2* gene encodes a mitochondrial‐targeted class 1 cysteinyl‐tRNA synthetase (CARS2; MIM*612800). Biallelic pathogenic variants in this gene have been identified as causative of the mitochondrial translation disorder combined oxidative phosphorylation deficiency 27 (MIM#616672). Eight unique *CARS2* gene variants have been previously reported in seven distinct individuals. They have all presented with infantile or childhood‐onset epileptic encephalopathy and developmental regression^9^, ^10^, ^11^, ^12^, ^13^ (Table 1).

**TABLE 1:** Summary of previously reported clinically relevant DNA variants in the *CARS2* gene, alongside their biochemical effects and clinical associations.

CASE	CASE 1	CASE 2	CASE 3	CASE 4	CASE 5	CASE 6	CASE 7	CASE 8
	This report	Kapoor^13^	Hu^23^	Wu^12^	Samanta^11^	Coughlin^10^	Hallman^9^	Hallman^9^
Age at presentation or symptom onset	16 days	9 years	6 months	2 years	Unknown (early onset; diagnosed with autism at 3 years)	5 weeks	9 years	5 years
Age of death or last reported examination	16 weeks	11 years	Unknown	4 years 7 months	13 years	3 years 10 months	28 years	18 years
Sex	Male	Male	Male	Unknown	Female	Female	Male	Female
Variants	c.655G>A, p.Ala219Thr; c.1478T>C, p.Phe493Ser (CH)	c.655G>A, p.Ala219Thr (Hom)	c.1426G>T, p.Gly476Arg (Hom)	c.323T>G, p.Phe108Cys; c.1036C>T, p.Arg346Trp (CH)	c.155T>G, p.Val52Gly; c.563C>T, p.Thr188Met (CH)	c649_651delGAG, p.Glu217del; c.752C>T, p.Pro251Leu (CH)	c.655G>A, p.Ala219Thr (Hom)	c.655G>A, p.Ala219Thr (Hom)
Brain MRI	6 weeks: Normal 14 weeks: The spectroscopy was abnormal with an increased lactate level and decreased NAA, in keeping with neuronal death.		Minor abnormalities	Brain atrophy	Global cerebral and cerebellar atrophy Very thin corpus callosum	Progressive microcephaly, including atrophy of the cortex and white matter Thin corpus callosum	Bilateral white matter lesions in the occipital lobe Global brain atrophy	
EEG	EEG at 4 weeks: Mildly abnormal EEG for age because of excess slowing for age and state and the presence of rare right occipital sharp transients. EEG at 6 weeks: Mildly abnormal EEG due to a mild excess of temporal sharp waves arising over either temporal region. The remainder of the background activity appeared within normal limits. 48 h video EEG at 9 weeks: An episode of stiff movements associated with increased blood pressure. No evidence of electrographic seizure activity. 48 h video EEG at 14 weeks: Background shows dysmaturity but is within reasonable limits for age. Rare, isolated interictal discharges over both the left and right posterior region, on this occasion more prominently over the left posterior region. Clinical events documented did not have any electrical correlate, and were not epileptiform.	Highly abnormal background with generalized spike‐and‐wave discharges		Seizures localized to bilateral frontal poles with temporal spikes			Generalized grouped spike and double spike wave complexes	Generalized grouped spike and double spike wave complexes White matter lesions in the left parietal lobe and brainstem
Seizures	No		Yes (refractory seizures)	Yes (generalized tonic–clonic seizures)	Yes (intractable epilepsy/status epilepticus; left occipital pseudoperiodic epileptiform discharges)	Yes (epileptic encephalopathy with intractable seizures, in the form of multifocal epileptiform discharges and frequent focal myoclonic and complex partial seizures)	Yes (severe myoclonic epilepsy)	Yes (severe myoclonic epilepsy)
Movement disorder	Dystonia with superimposed episodic dystonic crises associated with life threatening apnoea		Progressive myoclonus		Psychomotor regression	Complex movement disorder, including hypotonia and dystonia, chorea, myoclonus and abnormal movements	Progressive tetraparesis	Progressive tetraparesis
Developmental regression	Yes	Yes (neuroregression)	Yes (neuroregression)		Yes (significant developmental regression)	Yes (neurological regression)	Yes (cognitive decline)	Yes (cognitive decline)
Multisystem disease	No—normal ultrasound of abdomen, renal and liver function tests, hearing and ocular examination	No	No	No	Yes	Yes	Yes	Yes
mtRCE testing in muscle	Low complex I and IV activity	N/A	N/A	N/A	N/A	Low complex I and IV activity	N/A	N/A
mtRCE testing in skin fibroblasts	Normal	N/A	N/A	N/A	N/A	Low complex IV	N/A	N/A
Blue native gel separation in skin fibroblasts	Normal	N/A	N/A	N/A	N/A	N/A	N/A	N/A
Aminoacylation testing in skin fibroblasts	mt‐tRNA^Cys^ aminoacylation deficiency	N/A	N/A	Aminoacyl‐tRNA synthetase deficiency	N/A	N/A	N/A	N/A

*Note*: Asterisks reflect reports documenting minimal clinical information.

Abbreviations: CH, compound heterozygous; Het, heterozygous; Hom, homozygous; mtRCE, mitochondrial respiratory chain enzyme; N/A, either not applicable or not reported.

Here we present a New Zealand boy with European ancestry who had a severe neonatal presentation with movement disorder, severe laryngomalacia requiring tracheostomy and central apnoea resulting in early death. Whole‐exome sequencing revealed biallelic variants in the *CARS2* gene, thus expanding the phenotypic and genotypic spectrum of this rare mitochondrial disease.

## 2 CASE REPORT

The proband was the second child of a healthy nonconsanguineous European couple. His older sister was unaffected and otherwise healthy. He was born at term by normal delivery after an uneventful pregnancy. There were concerns about his feeding but he was discharged home from the postnatal ward to midwifery care at day three of life. He presented at day 16 to a peripheral hospital with blue episodes, stridor and choking on his feeds. He had poor weight gain and clear evidence of an abnormal suck and swallow. Apnoea was thought to be due to airway collapse with severe laryngomalacia, abnormal arytenoids and retroflexed epiglottis noted on nasal endoscopy. He underwent a supraglottoplasty at day 20 of life. He was commenced on nasogastric feeding and he had no further apnoea events. He began to focus, fix and was alert. He developed apnoea again at 6 weeks of age and underwent a revision supraglottoplasty. His apnoea persisted and despite a patent airway he developed an episode of severe respiratory acidosis. He was intubated and transferred to a centralized pediatric intensive care unit.

On arrival he had increased tone globally and was hyperreflexic in his lower limbs. He had no exaggerated startle. He had an abnormal breathing pattern at rest and unusual movements with stiffening, initially thought to be seizures, but without EEG correlate. He had an extensive neuro‐metabolic work‐up, including CSF biogenic mono‐amine metabolites and amino acids and lactate, which were normal. He had a multisystem review which was unremarkable. Brain MR imaging was initially normal with normal MR spectroscopy. He had repeat EEGs, including prolonged monitoring capturing clinical events, which were nonspecific and not suggestive of an underlying epileptic disorder (Table 1).

The proband was extubated at 6.5 weeks but continued to have multiple brief apnoeic events. He was again found to have laryngomalacia and a tracheostomy was placed at 7 weeks of age. Between events he had a normal breathing pattern but his tone remained increased and his suck and swallow dyscoordinated. As time progressed he developed increasing frequency of dystonic crises, captured on continuous video EEG as nonepileptiform. He had increasingly frequent pauses in his breathing in hospital, associated with bradycardia. He did not make any developmental progress and progressively lost visual regard.

Repeat MR imaging at 12 weeks showed normal brain volume and signal, including the brain stem, but low NAA:choline peak suggestive of neuronal cell death and an elevated lactate peak. Muscle biopsy revealed reduced mitochondrial respiratory chain enzyme (RCE) activity for cytochrome *c* oxidase (Complex IV) and NADH‐coenzyme Q_1_ oxidoreductase (Complex I) (Table S1). MtDNA sequencing of DNA extracted from muscle revealed homoplasmy for a polymorphism m.2628T>C in the *MT‐RNR2* gene, but was otherwise normal. At 14 weeks of age, redirection of care was established and a decision was made to allow a natural death. He died of respiratory failure due to central apnoea at 16 weeks of age, without a formal diagnosis.

## 3 MATERIALS AND METHODS

### 3.1 Whole‐exome sequencing

Trio whole‐exome sequencing (WES) of the patient's and parents' DNA was carried out postmortem using an Illumina HiSeq platform, as previously described^14^ and as detailed in the online Supplementary Methods.

### 3.2 Mitochondrial functional studies

Aminoacylation of the mitochondrial tRNA for cysteine (mt‐tRNA^Cys^) was analyzed as described previously.^10^, ^15^ Briefly, total RNA was extracted from the patient's skin fibroblasts using Trizol reagent (Life Technologies) according to the manufacturer's instructions, with the final pellet resuspended in 10 mM NaOAc at pH 5.0 and kept at 4°C to preserve the aminoacylation state. For the deacylated control, the pellet was resuspended in 200 mM Tris–HCl at pH 9.5 and incubated at 75°C for 5 min, followed by RNA precipitation and resuspension in 10 mM NaOAc at pH 5. Next, 5 μg of RNA was separated on a 6.5% polyacrylamide gel (19:1 acrylamide:bisacrylamide) containing 8 M urea in 0.1 M NaOAc pH 5.0 at 4°C and blotted onto a nylon transfer membrane (Hybond, GE). Following UV‐crosslinking, the membrane was hybridized with appropriate radiolabelled riboprobes, washed and imaged using a Typhoon PhosphorImager. Densitometric quantification was performed using ImageJ.

Spectrophotometric respiratory chain enzyme analysis was performed on post‐600 × *g* supernatants on a Cary 300 spectrophotometer using methods previously described for skeletal muscle^16^ and skin fibroblasts.^10^ Nondenaturing blue native gel electrophoresis was performed on solubilized mitochondrial inner membrane preparations with identification by colorimetric in gel activity staining assays as previously described.^10^, ^17^ The assembly of complex I was analyzed using a nondenaturing gel of the mitochondrial inner membrane fraction and the complexes identified after Western blotting using an antibody against NDUFS2 as previously described.^18^

## 4 RESULTS

### 4.1 Variant identification

**FIGURE 1:**
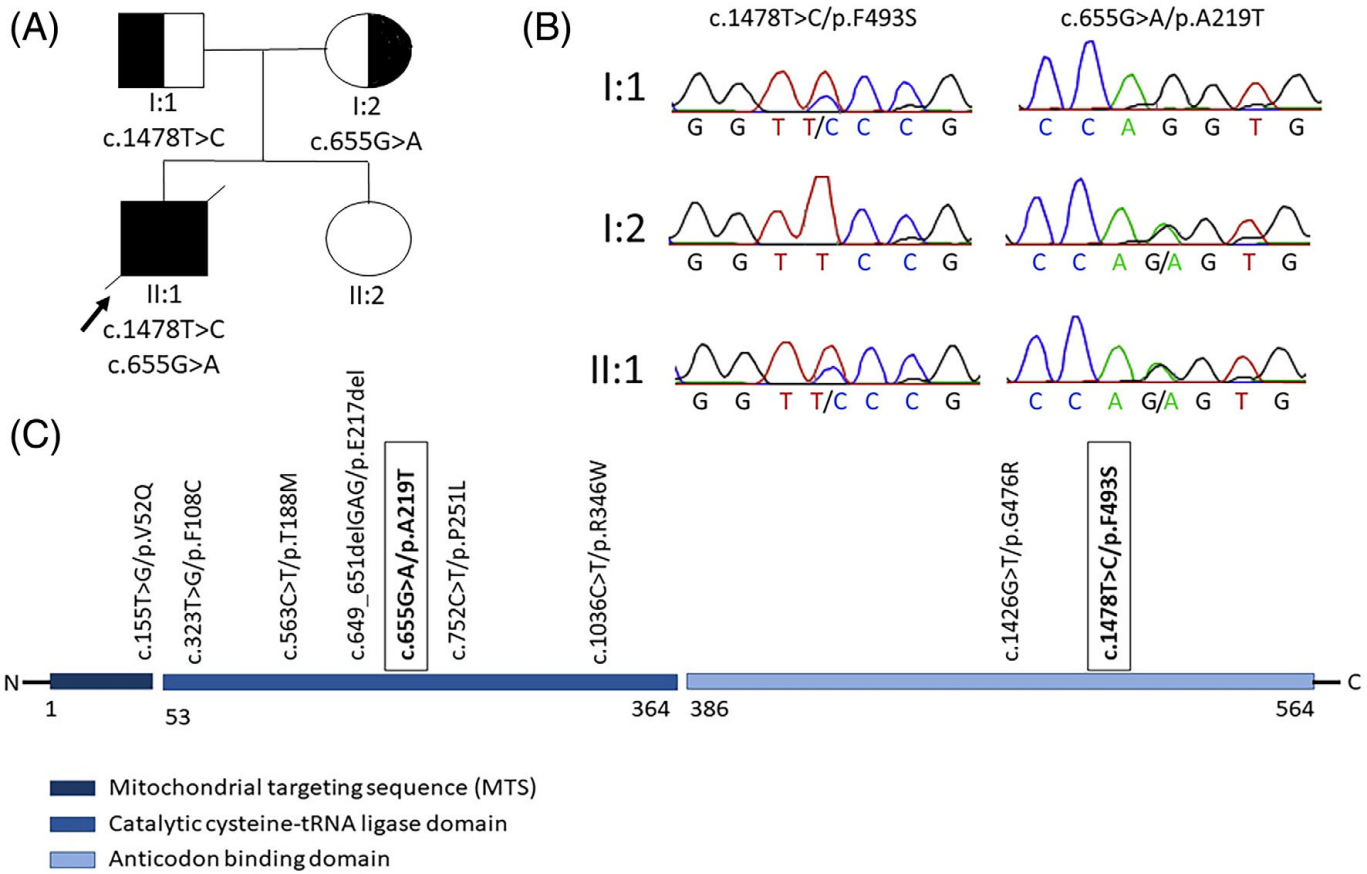
Summary of *CARS2* gene variants. (A) Pedigree showing the immediate family of the proband carrying the compound heterozygous variants in the *CARS2* gene (the unaffected sister was not sequenced). (B) Sanger sequencing electropherograms showing (1) c.1478T>C, p.Phe493Ser, and (2) c.655G>A, p.Ala219Thr in *CARS2*. (C) Visualization of protein locations of previously reported variants in the *CARS2* gene. Reported variants include 7 missense variants and 1 in‐frame deletion, disproportionately affecting the cysteine‐tRNA ligase domain. The variants documented in this report appear framed in bold.

Following variant filtering and prioritization (as detailed in the online Supplementary Information), two compound heterozygous single nucleotide variants in the *CARS2* gene were identified: a novel, paternally inherited NM_024537.4:c.1478T>C, p.Phe493Ser substitution and a maternally inherited c.655G>A substitution (NM_024537.4:c.655G>A, NP_078813.1:p.Ala219Thr, rs727505361; ClinVar accession ID VCV000180135.3; MAF = 0.00001266 (gnomAD v2.1.1)) (summarized in Figure 1A,B and Table S2). The c.1478T>C substitution affects exon 14 (of 15) at a highly conserved codon (with an averaged PhyloP 100 vertebrates base wise conservation score of 5.17201) in a cross‐vertebrate conserved amino acid (Clustal Omega alignment in Figure S1). It is a novel variant and the second reported to affect the CARS2 anticodon binding domain, replacing a hydrophobic phenylalanine with a polar uncharged serine (NP_078813.1:p.Phe493Ser). The previously reported c.655G>A substitution affects the 3′ nucleotide of exon 6 causing aberrant splicing. As established by Hallmann et al., it effectively removes exon 6 from the *CARS2* gene transcript, leading to an 84 nucleotide in‐frame deletion within a conserved sequence motif, compromising the stability of the acceptor end hairpin of CARS2.^9^ These variants were classified as variants of uncertain significance based on ACMG sequence variants interpretation guidelines due to their low population frequencies (PM2—Moderate), *in silico* predicted protein effects (as summarized in Table S2) (PP3—Supporting), and, for the c.655G>A variant, the previous association of the c.655G>A substitution to a movement disorder^9^ (PS4—Moderate).

### 4.2 Skin fibroblast aminoacylation studies

**FIGURE 2:**
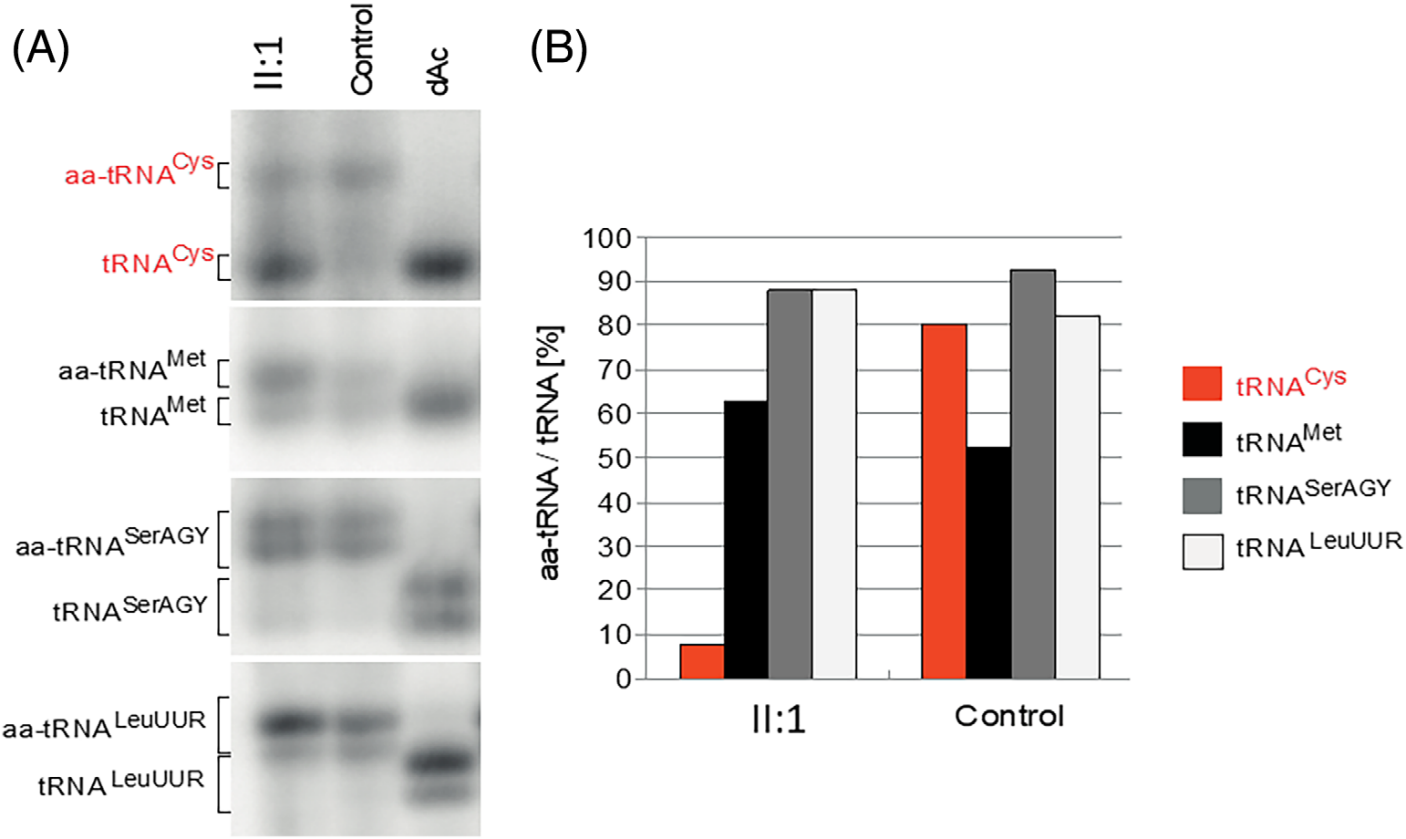
Characterization of the molecular defects of *CARS2* gene variants. (A) Northern blot of mitochondrial tRNA samples from proband primary skin fibroblasts (II:1), control NHDF‐Neo fibroblasts (Control), and control *in vitro* deacetylated NHDF‐Neo fibroblasts (dAc). Aminoacylated tRNAs are labeled aa‐tRNAs, whereas uncharged tRNAs are labeled as tRNAs. (B) Densitometric quantification of aa‐tRNA/tRNA (%) ratios in proband (II:1) and control NHDF‐Neo fibroblasts (Control). Values represent the sum of the single (tRNA^Cys^ and tRNA^Met^) or two (tRNA^SerAGY^ and tRNA^LeuUUR^) bands.

The functional impact of the *CARS2* variants on the mt‐tRNA^Cys^ was assessed by high‐resolution northern blotting of RNA isolated from patient primary skin fibroblasts. The ratio of aminoacylated and deacylated forms of mt‐tRNA^Cys^ was substantially decreased in patient primary skin fibroblasts compared to control human neonatal dermal (NHDF‐Neo) fibroblasts (7.8% in II:1 vs. 80.4% in the control), while no differences were observed in mt‐tRNAs tRNA^Met^ (62.8% in II:1 vs. 52.2% in the control), tRNA^SerAGY^ (88.0% in II:1 vs. 92.7% in the control) and tRNA^LeuUUR^ (88.0% in II:1 vs. 82.3% in the control) (Figure 2A,B).

### 4.3 Skin fibroblast respiratory chain enzyme analysis

Respiratory chain enzyme activities in the patient's primary skin fibroblasts were within normal limits. The blue native gel separation of the complexes with in‐gel activity staining showed normal activities for complexes I, II, IV, and V and normal assembly of complex V without pathological lower molecular weight F_1_ intermediates, as is sometimes noted with translation defects^10^, ^19^ (Figure S2, Table S3). The assembly of complex I was normal, without abnormal intermediates and a normal amount of holocomplex.

## 5 DISCUSSION

This is the first report of biallelic pathogenic variants in the *CARS2* gene (a novel pathogenic c.1478T>C variant in combination with a previously reported c.655G>A variant) resulting in neonatal onset neuroregression with progressive paroxysms of dystonia, apnoea and eventual death at 16 weeks of age.

The c.655G>A substitution, previously characterized by both Hallman et al. and Kapoor et al.,^9^, ^13^ affects an amino acid that is highly conserved in vertebrates^9^ and is responsible for a splicing defect that removes exon 6 and results in the deletion of 28‐amino acids in a surface loop of the CARS2 ligase catalytic domain. This specific loop stabilizes the acceptor end hairpin by making contacts at its G1‐C72 base pair and at the backbone of U73. The removal of these 28 amino acids is thus predicted to compromise acceptor hairpin stability and alter cysteine‐tRNA binding, substantially reducing aminoacylation activity.^9^ Interestingly, half of all reported individuals to date (four of eight) suffering from *CARS2*–related disease harbor at least one allele with this specific c.655G>A variant.^9^, ^13^

The novel variant c.1478T>C substitutes a phenylalanine with a serine at residue 493. This is the second reported variant affecting the CARS2 anticodon binding domain (Figure 1C). It affects a cross‐vertebrate conserved residue, replacing a hydrophobic with a polar uncharged residue, and may thus compromise the localized anticodon binding activity of CARS2.

A mitochondrial tRNA aminoacylation assay on tRNA^Cys^, tRNA^Met^, tRNA^SerAGY^, and tRNA^LeuUUR^ was performed in primary skin fibroblasts from the proband (Figure 2A,B), confirming that these biallelic variants likely result in the impairment of the vital aminoacylation function of CARS2.

Loss of mitochondrial protein function in the proband skeletal muscle was evidenced by decreased activity of respiratory chain complexes I and IV (Table S1). Only one of six previous reports of *CARS2*‐related disease also underwent a mitochondrial respiratory chain enzyme (mtRCE) analysis in skeletal muscle, which showed a tendency toward low activity of respiratory chain complexes I and IV, alongside incomplete assembly of complex V.^10^ This is consistent with Coughlin et al.'s observation that combined deficient activity of respiratory chain complexes may result from defects in the proteins required for mtDNA maintenance, transcription, or translation.^10^ The proband also underwent extensive testing of mitochondrial function in skin fibroblast cells, revealing no abnormality. Interestingly, apparently normal skin fibroblast mitochondrial function is often observed in mt‐aaRS related disorders, where only 50% of cell lines show functional defects,^20^ thus exemplifying the unusual behavior of pathogenic variants in *ARS2* genes in different tissues.

The disordered function of mt‐aaRSs results in a spectrum of phenotypes ranging from severe neurological disease to primary ovarian failure. Despite the seemingly vital role of these tRNA synthetase proteins in the translation of mtDNA encoded proteins, there is a propensity to a singular presentation with or without neurological involvement, often in the absence of the classical features of mitochondrial disease, such as multisystem involvement or lactic acidosis. The reason for this is not fully understood but may be due to some form of corrective mechanism in unaffected tissues.

*CARS2‐*related disease is characterized by severe neuroregression and epilepsy with nonspecific findings on brain imaging and no clear pattern of multisystem involvement or biochemical evidence of mitochondrial disease. Pathogenic variants in *RARS2*, the gene encoding the mitochondrial tRNA synthetase for arginine, lead to a similar neuroregressive clinical phenotype. Though most have striking pontocerebellar hypoplasia,^21^ a recent report describes two older patients without clear MR imaging abnormalities.^22^ Treatment refractory epilepsy, progressing to early death from three to 28 years of age, has been described in all reported cases of *CARS2*‐related disease (Table 1). In contrast, the proband reported here underwent five EEG recordings over the course of his short life with capture of clinical events and showed no clear evidence of epileptiform activity. This may be because he died prior to the onset of epileptic seizures. Complex movement disorders including dystonia, choreoathetoid movements and myoclonus have also been described previously in *CARS2*‐related disease.^10^, ^12^ Consistently, the proband had an early onset of a severe movement disorder with dystonia and associated apnoea that led to his death.

To date, there have been two reported cases of hearing and visual loss^9^ and one of liver disease associated with *CARS2* gene variants^10^ (Table 1). In addition, the neuroimaging reported in *CARS2*‐related disease to date is not typical of classical mitochondrial disease, with brain atrophy being the most frequently described feature.^9^, ^10^, ^11^, ^12^ In contrast, the only abnormality detected in the proband was a low NAA:choline ratio and elevated lactate on MR spectroscopy with normal imaging. The latter may be due to the early presentation, confirming that normal neuroimaging in the context of a severe neurological presentation does not exclude a mitochondrial disorder.

## 6 CONCLUSION

We have leveraged WES to identify and functionally confirm the pathogenicity of rare biallelic variants in the *CARS2* gene, which resulted in severe neonatal onset neuroregression, dystonia, progressive central apnoea, and early infantile death. This case expands the known genotypic and phenotypic spectrum of *CARS2*‐related disease while supporting the clinical utility and cost‐ and time‐saving nature of massively parallel sequencing technologies in the diagnosis of patients with mitochondrial disorders.

## AUTHOR CONTRIBUTIONS

Jessie C. Jacobsen, Russell G. Snell, Johan L. K. Van Hove, and Klaus Lehnert designed the experiments. Jessie C. Jacobsen and Klaus Lehnert performed the whole‐exome sequencing bioinformatic analysis. Jessie C. Jacobsen, Klaus Lehnert, and Jessie Poquérusse performed the variant interpretation analyses. Emma Glamuzina and Melinda Nolan clinically evaluated the patient. Juliet Taylor and Donald R. Love clinically confirmed the research results. Christopher A. Powell and Michal Minczuk performed the Northern *blot* analysis of mitochondrial tRNA *aminoacylation*. David R. Thorburn performed the respiratory chain enzyme analysis of skeletal muscle. Marisa W. Friederich and Johan L. K. Van Hove performed the respiratory chain enzyme analysis of primary skin fibroblasts. Jessie Poquérusse, Jessie C. Jacobsen, and Emma Glamuzina wrote the manuscript.

## FUNDING INFORMATION

J. C. J. was supported by the Neurological Foundation of New Zealand and a government‐funded Rutherford Discovery Fellowship administered by the Royal Society of New Zealand. The research was funded by The Neurological Foundation of New Zealand and The University of Auckland. D. R. T. was supported by the Australian National Health and Medical Research Council. C. A. P and M. M. were supported by The Medical Research Council, UK (MC_UU_00015/4 and MC_UU_00028/3). The authors confirm independence from the sponsors; the content of the article has not been influenced by the sponsors. This study was also supported by a grant from the National Institutes of Health, NIH U54NS078059 for the North American Mitochondrial Disease Consortium (NAMDC) (JVH). NADMC is part of the Rare Diseases Clinical Research Network (RDCRN), an initiative at the Office of Rare Diseases Research (ORDR), NCATS. This consortium is funded through collaboration between NCATS. Financial support was also received from the University of Colorado Foundation (mitochondrial research fund) and the Children's Hospital Colorado Foundation (Riders for Samantha) (JVH and MWF). The content is solely the responsibility of the authors and does not necessarily represent the official views of the National Institutes of Health. Funding sources had no role in the design or execution of the study, in the interpretation of data or the writing of the study.

## CONFLICT OF INTEREST

Michal Minczuk is a co‐founder, Scientific Advisory Board member and shareholder of Pretzel Therapeutics. JVH participated on the advisory board of Stealth Biotherapeutics. The other authors declare no conflicts of interest.

## ETHICS STATEMENT

Ethical approval was obtained by the Northern B Health and Disability Ethics Committee (12/NTB/59) prior to acquiring, sequencing and analyzing genetic information from the family. The work on the fibroblasts in Colorado was performed under an IRB‐approved protocol (COMIRB# 18‐1828). All procedures were performed in accordance with the ethical standards of the institutional and national responsible committees on human experimentation and with The Code of Ethics of the World Medical Association (*Declaration of Helsinki, 2013*).

## PATIENT CONSENT

Written informed consent to be included in the study was obtained from all participants.

## Supporting information


**Table S1.** Mitochondrial respiratory chain enzyme activities from the patient's skeletal muscle biopsy. The activities of respiratory chain complexes I, II, combined complex II–III and citrate synthase (CS) activity are expressed as nmol min^−1^ mg protein^−1^ while activities of complexes III and IV are shown as first order rate constants (expressed as nmol^−1^ min^−1^). Activities are also shown as ratios to the activity of CS and complex II. Values in bold underline are <20% of normal control mean and values in bold correspond to 20%–30% of normal control mean, corresponding to major and minor criteria in the Bernier diagnostic scheme.^14^
**Table S2.** Summary of the *CARS2* gene mutations revealed by whole‐exome sequencing alongside their *in silico* predicted effects.
**Table S3.** Mitochondrial respiratory chain enzyme activities from the patient's primary skin fibroblasts. The activities of respiratory chain complexes I, II, combined complex II–III and citrate synthase (CS) activity are expressed as nmol min^−1^ mg protein^−1^ while activities of complexes III and IV are shown as first order rate constants (expressed as nmol^−1^ min^−1^). Activities are also shown as ratios to the activity of CS and complex II. The values are also expressed as standard deviations (*Z*‐score) of the log transformed values of controls, which are normally distributed.
**Figure S1.** ClustalOmega multiple sequence alignment shows conservation of the amino acid phenylalanine at position of 493 of the CARS2 protein across 14 species representing the extended vertebrate subphylum. The positions of the first and last amino acid for each sequence are indicated numerically. An asterisk connotes amino acid identity across all vertebrate species analyzed, while a colon connotes amino acids exceeding a score of >0.5 in the PAM 250 matrix, and a period connotes amino acids scoring ≤ 0.5 in the PAM 250 matrix. The red p.F493S arrow points to the location of the paternally inherited CARS2 amino acid change identified in patient II:1.
**Figure S2.** Blue native polyacrylamide gel electrophoresis with in‐gel activity staining from the patient's primary skin fibroblasts. The activity of complexes I, II, IV, and V is shown by in‐gel activity staining following separation on a blue native polyacrylamide gel electrophoresis.

## Data Availability

Consent was not obtained to deposit full variant calling data publicly.
