# Supplementary material for: Severe neonatal onset neuroregression with paroxysmal dystonia and apnoea: Expanding the phenotypic and genotypic spectrum of CARS2‐related mitochondrial disease
Source: JIMD Rep. 2023 Jan 22;64(3):223–32. doi: 10.1002/jmd2.12360 (PMC10159863; doi:10.1002/jmd2.12360)
Supplement: Supplementary file 1 — Table S1. Mitochondrial respiratory chain enzyme activities from the patient's skeletal muscle biopsy. The activities of respiratory chain complexes I, II, combined complex II–III and citrate synthase (CS) activity are expressed as nmol min−1 mg protein−1 while activities of complexes III and IV are shown as first order rate constants (expressed as nmol−1 min−1). Activities are also shown as ratios to the activity of CS and complex II. Values in bold underline are <20% of normal control mean and values in bold correspond to 20%–30% of normal control mean, corresponding to major and minor criteria in the Bernier diagnostic scheme. 14 Table S2. Summary of the CARS2 gene mutations revealed by whole‐exome sequencing alongside their in silico predicted effects. Table S3. Mitochondrial respiratory chain enzyme activities from the patient's primary skin fibroblasts. The activities of respiratory chain complexes I, II, combined complex II–III and citrate synthase (CS) activity are expressed as nmol min−1 mg protein−1 while activities of complexes III and IV are shown as first order rate constants (expressed as nmol−1 min−1). Activities are also shown as ratios to the activity of CS and complex II. The values are also expressed as standard deviations (Z‐score) of the log transformed values of controls, which are normally distributed. Figure S1. ClustalOmega multiple sequence alignment shows conservation of the amino acid phenylalanine at position of 493 of the CARS2 protein across 14 species representing the extended vertebrate subphylum. The positions of the first and last amino acid for each sequence are indicated numerically. An asterisk connotes amino acid identity across all vertebrate species analyzed, while a colon connotes amino acids exceeding a score of >0.5 in the PAM 250 matrix, and a period connotes amino acids scoring ≤ 0.5 in the PAM 250 matrix. The red p.F493S arrow points to the location of the paternally inherited CARS2 amino acid change identified in patie [file JMD2-64-223-s001.docx]

Severe neonatal onset neuroregression with paroxysmal dystonia and apnoea: Expanding the phenotypic and genotypic spectrum of *CARS2*-related mitochondrial disease

**Supplementary Information**

**Supplementary Materials and Methods**

**DNA extraction**

High quality genomic DNA was extracted from whole peripheral blood obtained from the proband and his parents using a Gentra Puregene DNA Extraction kit (Qiagen) according to the manufacturer’s instructions.

**Whole-exome sequencing and analysis**

DNA from the proband and parents was sent for whole-exome sequencing (WES) (50X) at Otogenetics Corporation. Reads were mapped and aligned as previously described ^1^, and variants were called with the Genome Analysis Toolkit Best Practices, as described hereafter.

Libraries (PCR free) were prepared and sequencing performed using an Illumina HiSeq 2000/2500 instrument to generate 100bp paired-end reads. Reads were mapped with the Burrows-Wheeler aligner (v0.7.12) ^2^ to the 1000 Genomes Project human reference genome assembly GRCh37.p13/hg19 (February 2009 release). Optical and PCR duplicates were removed with Picard (v2.17.3) ^3^. Indels were realigned and base quality scores were recalibrated using the Genome Analysis Toolkit (v 3.4-0) ^4^. The alignments for the two children were combined with alignments from 437 unrelated individuals for joint variant discovery and genotyping with the GATK’s HaplotypeCaller and GenotypeGVCFs tools, followed by variant quality score recalibration according to the GATK’s Best Practices. Population frequency of SNV and indel variants were obtained from gnomAD releases 2.1.1 and 3.1.2 (ExAC) ^5^ and 1000 Genomes Project ^6^. WGS alignments were viewed using the Integrated Genomics Viewer (IGV, v2.3.55) ^7^.

**PCR and Sanger sequencing**

Variants identified in the *CARS2* gene were validated by PCR followed by Sanger sequencing. Primers were designed using Primer-BLAST ^8^. For NM_024537.4: c.1478T>C, the forward primer 5’ CATCCACAATCCACAGTCTCC 3’ and the reverse primer 5’ GGCACTCACCTTGATGTTGAT 3’ generated a 268 bp product encompassing the mutation. For NM_024537.4: c.655G>A, the forward primer 5’ TGCTGATATTCAGACTGAGAAGTG 3’ and the reverse primer 5’ CTGGAGCCATCCACAGAAAC 3’ generated a 301 bp product encompassing the mutation. All PCR products were generated using the Expand High Fidelity PCR System (Roche) with the following thermocycler conditions: 5 min initial denaturation at 94°C, with 30 cycles of 30-sec denaturation at 94°C, 30 sec of annealing at 55°C, and 45 sec of extension at 72°C, followed by 7 min of final extension at 72°C. Sanger sequencing was performed by the Genomics Centre, Auckland Science Analytical Services, The University of Auckland, New Zealand. Sanger sequencing data were viewed using Geneious (v8.1.5) (<http://www.geneious.com>).

**ClustalOmega multiple sequence alignment**

The ClustalOmega web server ([https://www.ebi.ac.uk/Tools/msa/clustalo/](https://www.ebi.ac.uk/Tools/msa/clustalo/v); version 1.2.4) was used to perform multiple alignment of the full-length protein sequences from fourteen species representing the vertebrate subphylum using default parameters.

**Supplementary Results**

**Variant identification**

101,876 variants were discovered by WES in the parents and the proband. Variants exceeding minor allele frequencies (MAF) of 0.01 in the gnomAD ^5^ or 1000 Genomes Project ^6^ population databases, or 0.05 in our in-house cohort of 437 individuals were excluded from further consideration. Of the 3,239 variants retained by the population frequency filters, 525 were predicted by VEP to have high-impact consequences in the proband. Of these, 403 were excluded because they occurred in genotypes or haplotypes shared with one or both parent(s). The 122 retained variants were prioritised based on their likelihood of having a highly deleterious consequence as deduced based on the gene’s pLI score ^9^, Variant Effect Predictor (VEP) consequence annotations ^10^ and *in silico* predictions by SIFT ^11^, PolyPhen2 ^12^ and CAROL ^13^ functional pathogenicity prediction algorithms. These underwent a final round of variant-by-variant prioritization according to a number of biologically informed heuristics incorporating previously established phenotypic or disease association and relevant biochemical/biological function.

In the process, among the highest confidence variants, a homozygous stop-gain variant c.1246G>A/p.Arg416* (rs183765686; MAF = 0.008118) was identified in *SLC49A3*, an atypical member of the solute carrier (SLC) family, but was excluded based on the lack of phenotypic overlap with the proband’s presentation and its presence in homozygous form in two individuals in gnomAD’s variant database. A compound heterozygous variant consisting of c.901G>A/p.Arg301Trp (novel) and c.318G>A/p.Asn106Asn (rs34278797; MAF = 0.018) in *GALNS*, associated with the lysosomal storage disease mucopolysaccharidosis, type IVA, was also identified but rejected due to lack of phenotypic, and specifically neurological, overlap with the proband’s presentation.

**Supplementary Table 1.** Mitochondrial respiratory chain enzyme activities from the patient’s skeletal muscle biopsy. The activities of respiratory chain complexes I, II, combined complex II-III and citrate synthase (CS) activity are expressed as nmol.min^-1^.mg protein^-1^ while activities of complexes III and IV are shown as first order rate constants (expressed as nmol^-1^.min^-1^). Activities are also shown as ratios to the activity of CS and complex II. Values in bold underline are <20% of normal control mean and values in bold correspond to 20-30% of normal control mean, corresponding to major and minor criteria in the Bernier diagnostic scheme ^14^.

|  | Activity (controls) | Ratio enzyme/CS | Ratio enzyme/complex II |
| --- | --- | --- | --- |
|  |  |  |  |
| Complex I | 16 (19-72) | **89 (222-474)** | 356 (599-1345) |
| Complex II | 46 (26-62) | 250 (280-382) | NA |
| Complex III | 36 (13-51) | 198 (72-402) | 789 (205-1066) |
| Complex II-III | 33 (30-76) | 179 (280-501) | 716 (750-1422) |
| Complex IV | **1.5 (3.3-9.1)** | **20 (39-59)** | **33 (120-182)** |
| Citrate synthase | 83 (85-179) | NA | NA |

**Supplementary Table 2.** Summary of the *CARS2* gene mutations revealed by whole-exome sequencing alongside their *in silico* predicted effects.

| **Chromosomal DNA variant**  **(GRCh37)** | **CDS variant** | **Protein variant** | **Inheritance** | **rs; MAF** | **Effect** | **SIFT score** | **PolyPhen2 score** | **Distance to splice site (bp)** |
| --- | --- | --- | --- | --- | --- | --- | --- | --- |
| 13:g.111294807A>G | NM_024537.4: c.1478T>C | NP_078813.1:  p.Phe493Ser | Paternal | Novel | Missense | 0.03 (deleterious) | 0.926 (probably damaging) | 62 |
| 13:g.111335398C>T | NM_024537.4: c.655G>A | NP_078813.1:  p.Ala219Thr | Maternal | rs727505361;  MAF = 0.00001266 (gnomAD) | Missense, splice site | 0.58 (tolerated) | 0.015 (benign) | 1 |
|  |  |  |  |  |  |  |  |  |

CDS: coding sequence; MAF: minor allele frequency.

**Supplementary Table 3.** Mitochondrial respiratory chain enzyme activities from the patient’s primary skin fibroblasts. The activities of respiratory chain complexes I, II, combined complex II-III and citrate synthase (CS) activity are expressed as nmol.min^-1^.mg protein^-1^ while activities of complexes III and IV are shown as first order rate constants (expressed as nmol^-1^.min^-1^). Activities are also shown as ratios to the activity of CS and complex II. The values are also expressed as standard deviations (Z-score) of the log transformed values of controls, which are normally distributed.

|  | Activity (controls)  nmol.min^-1^.mg protein^-1^ | SD | Ratio enzyme/CS | SD | Ratio enzyme/complex II | SD |
| --- | --- | --- | --- | --- | --- | --- |
|  |  |  |  |  |  |  |
| Complex I | 123 (49-131) | +1.2 | 310 (145-396) | +1.3 | 528 (237-754) | +0.9 |
| Complex II | 233 (131-364) | +0.1 | 587 (297-863) | +0.1 | NA | NA |
| Complex III | 31.1 (8-29.2) | +1.9 | 78 (19-65) | +2.0 | 134 (36-114) | +2.0 |
| Complex II-III | 135 (62-159) | +0.8 | 340 (131-376) | +1.0 | 580 (263-1100) | +0.3 |
| Complex IV | 8.6 (2.2-7.1) | +1.8 | 22 (6-23) | +1.9 | 37 (12-35) | +1.9 |
| Citrate synthase | 396 (254-554) | +0.0 | NA | NA | NA | NA |

**Supplementary Figure 1.** ClustalOmega multiple sequence alignment shows conservation of the amino acid phenylalanine at position of 493 of the CARS2 protein across fourteen species representing the extended vertebrate subphylum. The positions of the first and last amino acid for each sequence are indicated numerically. An asterisk connotes amino acid identity across all vertebrate species analysed, while a colon connotes amino acids exceeding a score of > 0.5 in the PAM 250 matrix, and a period connotes amino acids scoring =< 0.5 in the PAM 250 matrix. The red p.F493S arrow points to the location of the paternally inherited CARS2 amino acid change identified in patient II:1.


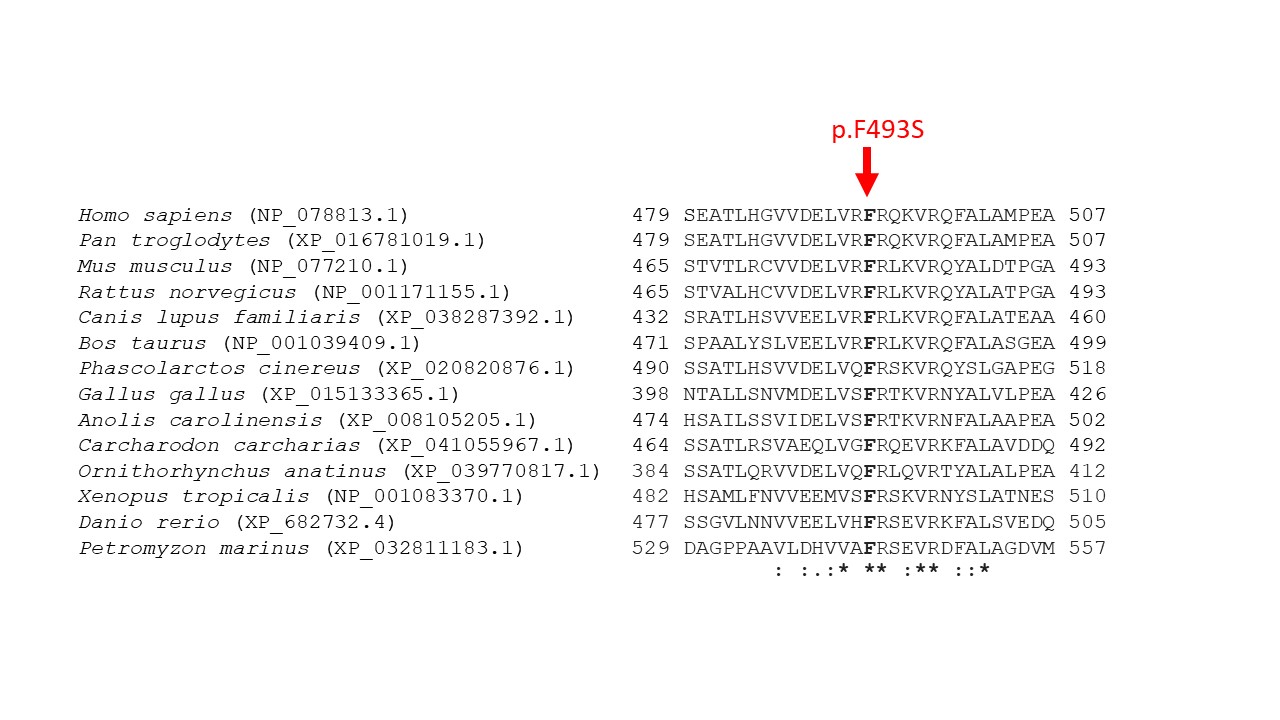


**Supplementary Figure 2.** Blue native polyacrylamide gel electrophoresis with in-gel activity staining from the patient’s primary skin fibroblasts. The activity of complexes I, II, IV, and V is shown by in-gel activity staining following separation on a blue native polyacrylamide gel electrophoresis.

**Control**

**Control**

**I**

**IV**

**V**

**II**

**II:1**

**II:1**


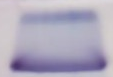

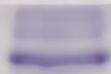

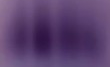

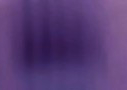

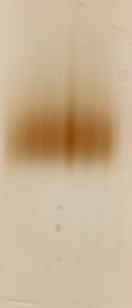

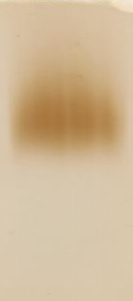

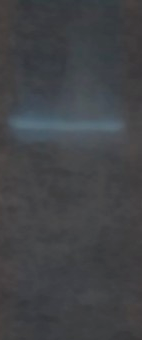

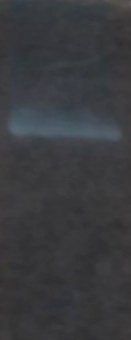


**References**

1. Poquérusse J, Whitford W, Taylor J, et al. Novel PRMT7 mutation in a rare case of dysmorphism and intellectual disability. *J Hum Genet.* 2022. 67, 19–26.

2. Li H, Durbin R. Fast and accurate short read alignment with Burrows-Wheeler transform. *Bioinformatics.* 2009. 25, 1754–1760.

3. Broad Institute. Picard tools. *https://broadinstitute.github.io/picard/* (2016). Available at: https://broadinstitute.github.io/picard/%5Cnhttp://broadinstitute.github.io/picard/.

4. Van der Auwera GA, Carneiro MO, Hartl C, et al. From FastQ Data to High-Confidence Variant Calls: The Genome Analysis Toolkit Best Practices Pipeline. *Curr Protoc Bioinformatics.* 2013. 43(1110):11.10.1-11.10.33.

5. Lek M, Karczewski KJ, Minikel EV, et al. Analysis of protein-coding genetic variation in 60,706 humans. *Nature.* 2016. 536, 285–291.

6. 1000 Genomes. A map of human genome variation from population-scale sequencing. *Nature.* 2010. 467, 1061–1073.

7. Thorvaldsdóttir H, Robinson JT, Mesirov JP. Integrative Genomics Viewer (IGV): High-performance genomics data visualization and exploration. *Brief. Bioinform.* 2013. 14, 178–192.

8. Ye J, Coulouris G, Zaretskaya I, et al. Primer-BLAST: a tool to design target-specific primers for polymerase chain reaction. *BMC Bioinformatics.* 2012. 13, 134.

9. Samocha KE, Robinson EB, Sanders SJ, et al*.* A framework for the interpretation of de novo mutation in human disease. *Nat Genet.* 2014. 46, 944–950.

10. McLaren W, Gil L, Hunt SE, et al. The Ensembl Variant Effect Predictor. *Genome Biol.* 2016. 17, 122.

11. Ng PC, Henikoff S. SIFT: Predicting amino acid changes that affect protein function. *Nucleic Acids Res.* 2003. 31, 3812–3814.

12. Adzhubei L, Jordan DM, Sunyaev SR. Predicting functional effect of human missense mutations using PolyPhen-2. *Curr Protoc Hum Genet.* 2013. 7, 7.20.

13. Lopes MC, Joyce C, Ritchie GRS, et al. A combined functional annotation score for non-synonymous variants. *Hum Hered.* 2012. 73, 47–51.

14. Bernier FP, Boneh A, Dennett X, et al. Diagnostic criteria for respiratory chain disorders in adults and children. *Neurology*. 2002. 59(9):1406-1411.
